# Phylodynamics of HIV-1 Subtype C Epidemic in East Africa

**DOI:** 10.1371/journal.pone.0041904

**Published:** 2012-07-27

**Authors:** Edson Oliveira Delatorre, Gonzalo Bello

**Affiliations:** Laboratório de AIDS & Imunologia Molecular, Instituto Oswaldo Cruz, Rio de Janeiro, Brazil; University of Florida, United States of America

## Abstract

The HIV-1 subtype C accounts for an important fraction of HIV infections in east Africa, but little is known about the genetic characteristics and evolutionary history of this epidemic. Here we reconstruct the origin and spatiotemporal dynamics of the major HIV-1 subtype C clades circulating in east Africa. A large number (*n* = 1,981) of subtype C *pol* sequences were retrieved from public databases to explore relationships between strains from the east, southern and central African regions. Maximum-likelihood phylogenetic analysis of those sequences revealed that most (>70%) strains from east Africa segregated in a single regional-specific monophyletic group, here called C_EA_. A second major Ethiopian subtype C lineage and a large collection of minor Kenyan and Tanzanian subtype C clades of southern African origin were also detected. A Bayesian coalescent-based method was then used to reconstruct evolutionary parameters and migration pathways of the C_EA_ African lineage. This analysis indicates that the C_EA_ clade most probably originated in Burundi around the early 1960s, and later spread to Ethiopia, Kenya, Tanzania and Uganda, giving rise to major country-specific monophyletic sub-clusters between the early 1970s and early 1980s. The results presented here demonstrate that a substantial proportion of subtype C infections in east Africa resulted from dissemination of a single HIV local variant, probably originated in Burundi during the 1960s. Burundi was the most important hub of dissemination of that subtype C clade in east Africa, fueling the origin of new local epidemics in Ethiopia, Kenya, Tanzania and Uganda. Subtype C lineages of southern African origin have also been introduced in east Africa, but seem to have had a much more restricted spread.

## Introduction

Human immunodeficiency virus type 1 (HIV-1) sequences belonging to the pandemic group M are classified into nine subtypes (A–D, F–H, J, and K), six sub-subtypes (A1–A4, and F1–F2), and a variety of inter-subtype recombinant forms (Los Alamos HIV sequence database: http://hiv-web.lanl.gov/). Subtype C is the most prevalent variant, accounting for nearly half (48%) of all global infections [1]. This high prevalence is due to the predominance of subtype C in southern Africa, east Africa and India, with further infections in central Africa and Brazil.

Subtype C accounts for >95% of HIV infections in all southern African countries [1]. Several studies showed that subtype C sequences from neighboring southern African nations display a great degree of phylogenetic intermixing with no evidence of significant geographical clustering [2], [3], [4], [5], [6], [7], indicating a largely unrestricted viral movement across the entire subcontinent. A more recent phylogenetic study revealed that after sequential pruning of ambiguously positioned taxa 10 strongly supported subtype C clusters becomes apparent in southern Africa, showing that the geographic subdivision of subtype C viruses circulating in this region is higher than expected by chance [8]. Most subtype C clusters identified, however, circulate in more than one southern African country and all four countries analyzed (Botswana, Malawi, South Africa and Zambia) comprise strains from multiple clusters. Thus, HIV epidemics in southern African countries are probably the result of the introduction and circulation of multiple subtype C strains with a variable level of local and regional dissemination.

In contrast to the southern African region, the prevalence of HIV-1 subtype C clade displays a great variation among eastern African countries. Subtype C reaches high prevalence in Burundi (>80%) [9], [10], Djibouti (>70%) [11] and Ethiopia (>95%) [12], [13], [14], [15], medium prevalence in Tanzania (20–40%) [16], [17], [18], [19], [20], and relatively low prevalence in Rwanda (14%) [21] and Uganda (<5%) [22], [23], [24], [25], [26], [27], [28]. Subtype C also accounts for a minor fraction (<15%) of HIV infections in western [29], [30], [31], coastal [28], [32], [33], [34] and central [28], [35], [36], [37] regions of Kenya; but displays a much higher frequency (25–50%) in some cities of the northern region that borders Ethiopia [38], [39].

Little is known about the genetic characteristics of HIV-1 subtype C strains circulating in east Africa. Previous studies showed that two genetically different subtype C strains designated C and C′, have been co-circulating in roughly similar prevalence and among the same risk groups and geographical areas in Ethiopia [13], [15], [40]. A recent study of Thomson and Fernández-García [8] revealed that the Ethiopian-C clade corresponds to one subtype C cluster also found in other east African countries including Burundi, Djibouti, Kenya, and Uganda; while the Ethiopian-C′ clade was assigned to an independent cluster associated to southern Africa. Other studies performed in Kenya showed that subtype C samples from this country are not concentrated in a single cluster, but distributed in several independent lineages associated to sequences from both east and southern Africa [34], [39]. Despite these previous studies, we still have an incomplete understanding of the number, onset date, and migration pattern of the distinct HIV-1 subtype C lineages circulating in the eastern African region.

To obtain a more comprehensive picture of the spatiotemporal dynamics of the HIV-1 subtype C epidemic in east Africa, we analyzed a large number of subtype C *pol* sequences sampled from the east (Burundi, Ethiopia, Kenya, Tanzania and Uganda), southern (Botswana, Malawi, Mozambique, South Africa, Zambia and Zimbabwe) and central (Angola and Democratic Republic of Congo) African regions over a time period of 25 years (1986–2010).

## Materials and Methods

### Sequence dataset

HIV-1 subtype C *pol* sequences from east, southern, and central African countries, that matched the selected genomic region (nt 2253–3272 relative to HXB2 clone) were retrieved from the Los Alamos HIV Database (http://hiv.lanl.gov). Countries were grouped in geographical regions according to the classification proposed by Hemelaar *et al* [1]. In order to improve the accuracy of phylogenetic inference only sequences from antiretroviral therapy naïve individuals were selected. The subtype assignment of all sequences was confirmed by the REGA HIV subtyping tool v.2 [41] and by maximum likelihood (ML) phylogenetic analysis (see below) with HIV-1 subtype reference sequences. Those sequences with incorrect subtype C classification, sequences containing frame-shift mutations or deletions, multiple sequences from the same individual and those sequences from countries poorly represented (<5 sequences) were removed. This resulted in a final dataset of 1,981 HIV-1 subtype C *pol* sequences sampled from 12 different African countries (Table 1). Sequences were aligned using the CLUSTAL X program [42] and alignment is available from the authors upon request.

**Table 1:** HIV-1 subtype C sequences.

African region	Country	*N*	Sampling date
Central	Angola	31	2001–2010
	Democratic Republic of Congo	22	2002–2007
Southern	Botswana	70	2001
	Malawi	46	2002
	Mozambique	101	2002–2004
	South Africa	1,031	1999–2009
	Zambia	150	1998–2008
	Zimbabwe	178	2007
East	Burundi	92	2002
	Ethiopia	102	1986–2003
	Kenya	39	1991–2007
	Tanzania	81	1997–2009
	Uganda	38	1990–2010

### Substitution saturation and likelihood mapping analyses

Substitution saturation was evaluated by plotting the estimated number of transitions and transversions against genetic distance for each pairwise comparison in our alignment of 1,981 HIV-1 subtype C *pol* sequences using DAMBE program [43]. The phylogenetic signal in the *pol* dataset was investigated with the likelihood mapping method [44] by analyzing 10,000 random quartets. Likelihood mapping was performed with TREE-PUZZLE program [45] using the online web platform Phylemon 2.0 [46].

### Phylogenetic analysis

ML phylogenetic trees were inferred under the GTR+I+Γ_4_ nucleotide substitution model, selected using the jModeltest program [47]. ML tree was reconstructed with PhyML program [48] using an online web server [49]. Heuristic tree search was performed using the SPR branch-swapping algorithm and the reliability of the obtained topology was estimated with the approximate likelihood-ratio test (*aLRT*) [50] based on the Shimodaira-Hasegawa-like procedure. The ML trees were visualized using the FigTree v1.3.1 program [51].

### Characterization of intrasubtype C/C′ recombinant sequences

Putative intrasubtype C/C′ recombinant sequences in Ethiopia were identified by Bootscanning using Simplot version 3.5.1 [52], following the same procedure described by Pollakis *et al* [40]. Bootstrap values supporting branching with reference sequences were determined in Neighbor-Joining (NJ) trees constructed using the K2-P nucleotide substitution model, based on 100 re-samplings, with a 300 bp sliding window moving in steps of 10 bases.

### Analysis of spatiotemporal dispersion pattern

The evolutionary rate (*μ*, units are nucleotide substitutions per site per year, subst./site/year), the age of the most recent common ancestor (*T*_mrca_, years), and the spatial dynamics of major subtype C clades from east Africa were jointly estimated using the Bayesian Markov Chain Monte Carlo (MCMC) approach implemented in the BEAST software package v1.6.2 [53], [54]. Analyses were performed using the GTR+I+Γ_4_ nucleotide substitution model, an uncorrelated Lognormal relaxed molecular clock model [55], a Bayesian Skyline coalescent tree prior [56], and a discrete phylogeographic model in which all possible reversible exchange rates between locations were equally likely [57]. Two separate MCMC chains were run for 4×10^8^ generations and adequate chain mixing was checked by calculating the effective sample size (ESS) after excluding an initial 10% for each run using program TRACER v1.4 [58]. MCMC runs converged to almost identical values and combined estimates showed ESS values >200. Maximum clade credibility (MCC) trees were summarized from the posterior distribution of trees with TreeAnnotator and visualized with FigTree v1.3.1. Migratory events were summarized using the cross-platform SPREAD application [59].

## Results

### Phylogenetic analysis

A large dataset of HIV-1 subtype C *pol* sequences (*n* = 1,981) downloaded from the Los Alamos HIV Database (http://hiv.lanl.gov) was used to characterize the relationship between subtype C sequences sampled from east, central and southern African countries. The transition/transversion *vs* divergence graphics showed that both type of nucleotide substitutions increase linearly with the genetic distance, with transitions being higher than transversions (Figure S1a), thus indicating no substitution saturation in our alignment. While, the likelihood-mapping analysis showed that most (90%) of the randomly chosen quartets from the HIV-1 subtype C alignment were equally distributed in the three corners of the likelihood map (Figure S1b), indicating a strong tree-like phylogenetic signal in the data. Both analyses indicate that the HIV-1 subtype C *pol* dataset used in this study contains enough evolutionary information for reliable phylogenetic and molecular clock inferences.

**Figure 1:**
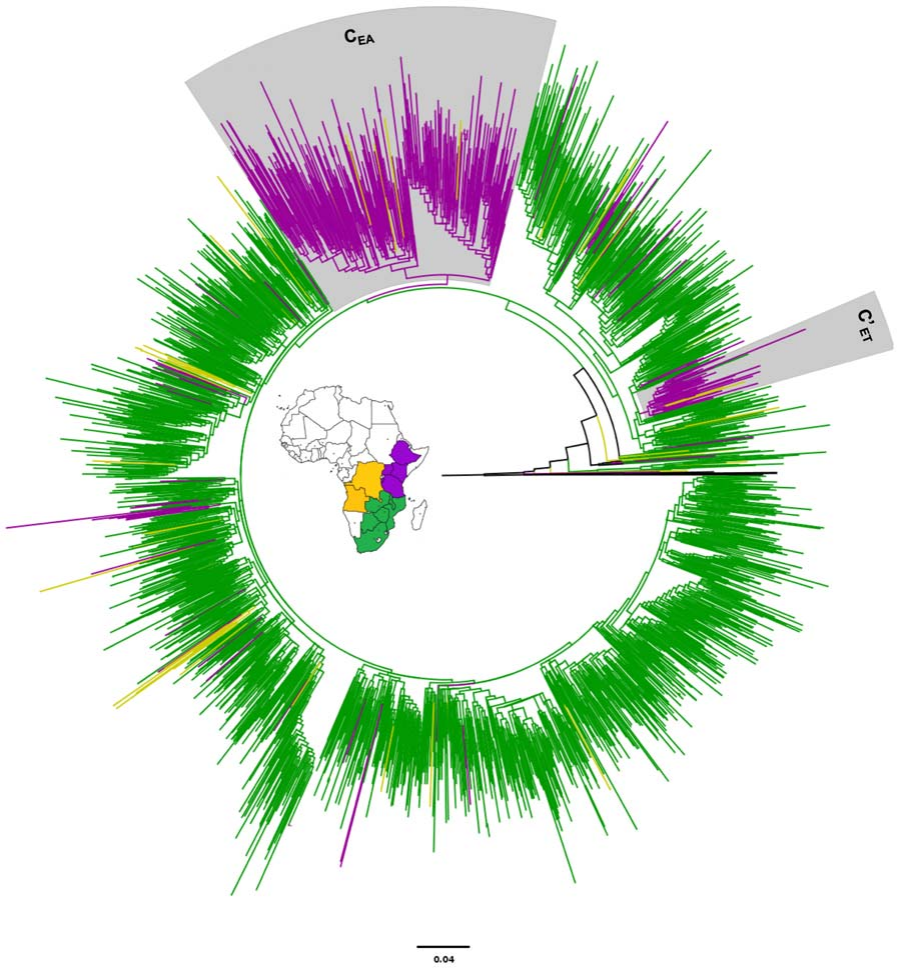
Maximum likelihood phylogenetic tree based on 1,981 HIV-1 subtype C *pol* (∼1,000 pb) sequences. Sequences were sampled at different countries from the east (*n* = 352), central (*n* = 53) and southern (*n* = 1,576) African regions shown in Table 1. The color of branches represents the geographic region from where the subtype C sequences originated, according to the map given in the figure. The boxes highlight the position of the major east African subtype C lineages. The tree was rooted using HIV-1 subtype A1 and D reference sequences (black branches). Horizontal branch lengths are drawn to scale with the bar at the bottom indicating nucleotide substitutions per site.

The ML phylogenetic analysis revealed that most (73%) subtype C sequences from east Africa branched within a highly supported (*aLRT* = 0.93) monophyletic cluster, here called C_EA_, that contains sequences from all five east African countries analyzed (Figure 1). Notably, the C_EA_ clade comprises a minor proportion (9%) of the 54 sequences from central Africa, but none of the 1,576 sequences from southern Africa here included. A minor fraction (11%) of subtype C sequences from east Africa branched in a second well supported (*aLRT* = 0.94) monophyletic cluster that comprises sequences from Ethiopia, and corresponds to the so called Ethiopian-C′ (C′_ET_) clade (Figure 1). The remaining subtype C east African sequences (16%) were distributed in several independent lineages of small size (*n*≤5 sequences) that were intermixed among strains from southern African countries (Figure 1).

**Figure 2:**
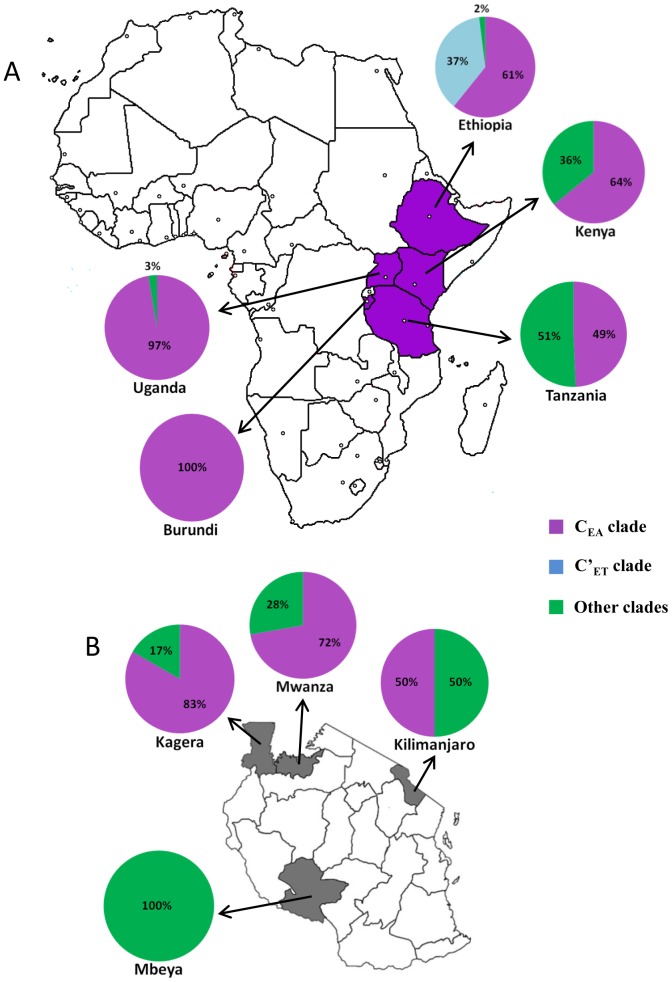
Geographic distribution of HIV-1 subtype C clades in east Africa. a) Map of Africa showing the frequency of distinct HIV-1 subtype C clades across the five countries from the east region here studied (Burundi, Ethiopia, Kenya, Uganda and Tanzania). b) Map of Tanzania showing the frequency of distinct HIV-1 subtype C clades across different country regions where patients included in the present study resided (Kagera, Mwanza, Kilimanjaro and Mbeya). The legend for the colors on graphics is shown on the right.

The analysis of sequence distribution among clades by country of origin revealed three different patterns within east Africa represented by Burundi/Uganda, Ethiopia and Kenya/Tanzania (Figure 2a). All or most subtype C strains circulating in Burundi and Uganda belong to the major clade C_EA_. Subtype C strains from Ethiopia, by contrast, were mainly distributed into clades C_EA_ (61%) and C′_ET_ (37%). Finally, about 64% of subtype C sequences from Kenya and 49% from Tanzania branched within the major clade C_EA_, while the remaining sequences were distributed in the multiple minor clades of southern African origin. Such geographical variation in the prevalence of different subtype C clades could be also observed at a more local scale in Tanzania (Fig. 2b). In the Kagera and Mwanza regions (north), most (>70%) subtype C strains belong to the C_EA_ clade. In the Kilimanjaro region (northeast), sequences from both the C_EA_ and “southern African” clades reach a roughly similar prevalence. In the Mbeya region (southwest), only “southern African” clades were detected.

### Migration pattern of HIV-1 C_EA_ clade

A closer inspection of the HIV-1 C_EA_ clade showed that sequences from Burundi occupies the most basal position in the clade (Figure S2), thus suggesting that Burundi was the most probable epicenter of dissemination of this subtype C lineage. The migration pattern of the C_EA_ lineage was reconstructed using a Bayesian statistical framework that allows ancestral reconstruction of the locations at the interior nodes of Bayesian tree while accommodating phylogenetic uncertainty. Sequences with no information about sampling date (*n* = 2), sequences with unexpectedly long branches in the phylogenetic analysis (*n* = 10), and Ethiopian sequences with evidence of intra-subtype recombination (*n* = 8, see below) were excluded from this analysis. This resulted in a final dataset of 236 sequences (Burundi = 92, Ethiopia = 47, Kenya = 24, Tanzania = 40, and Uganda = 33) sampled between 1990 and 2010.

**Figure 3:**
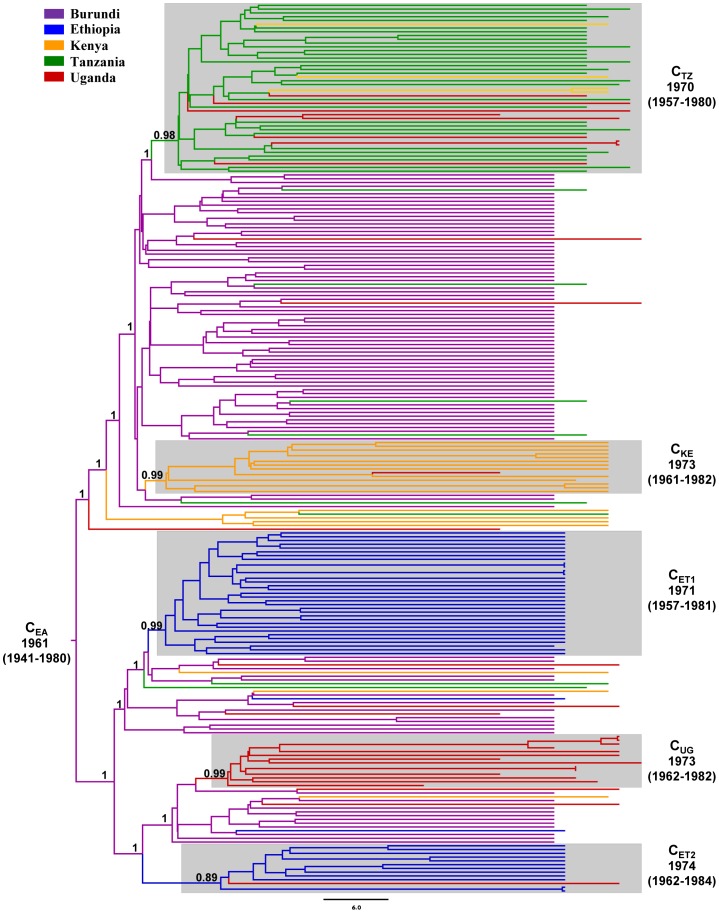
Time-scaled Bayesian MCC tree of the HIV-1 C_EA_ lineage. Branches are colored according to the most probable location state of their descendent nodes. The legend for the colors is shown on the left. The state posterior probability is indicated only at key nodes. The boxes highlight the position of the major country-specific sub-clades detected in our study. The median age (with 95% HPD interval in parentheses) of those country-specific sub-clades is shown. Horizontal branch lengths are drawn to scale with the bar at the bottom indicating years. The tree was automatically rooted under the assumption of a relaxed molecular clock.

**Figure 4:**
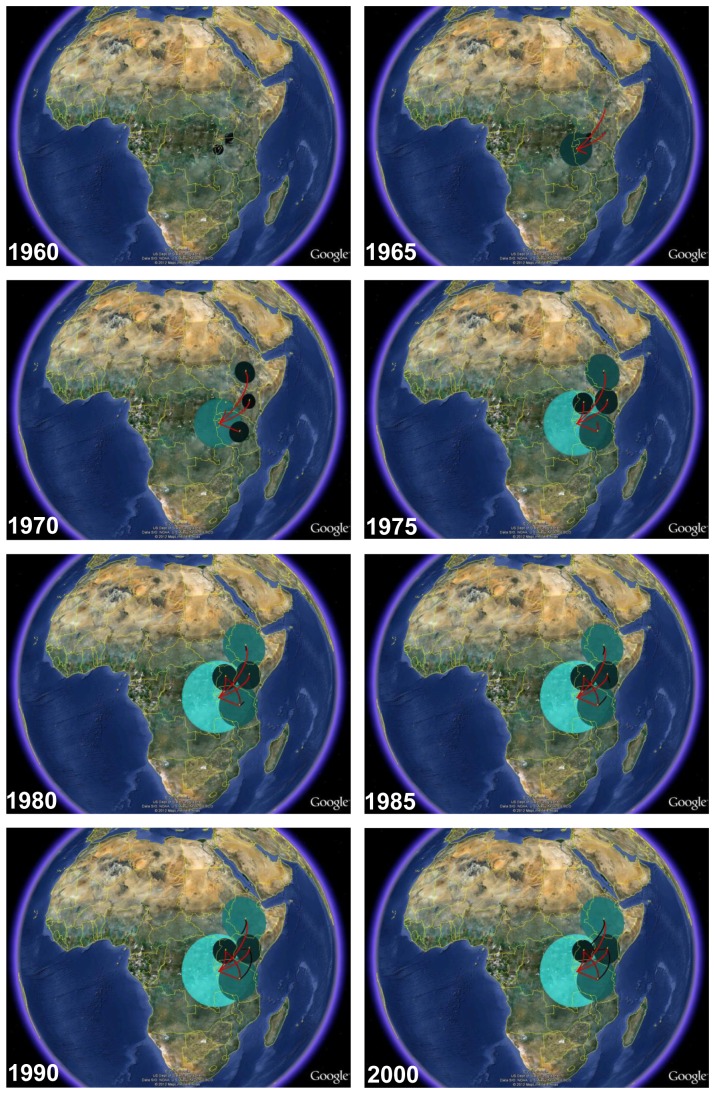
Spatiotemporal dynamic of HIV-1 C_EA_ clade dissemination in east Africa. We provide snapshots of the dispersal pattern for the years 1960, 1965, 1970, 1975, 1980, 1985, 1990 and 2000. Lines between locations represent branches in the Bayesian MCC tree along which location transition occurs. Location circle diameters are proportional to square root of the number of Bayesian MCC branches maintaining a particular location state at each time-point. The white-green color gradient informs the relative age of the transitions (older-recent). The maps are based on satellite pictures made available in Google^™^ Earth (http://earth.google.com).

The Bayesian MCC tree supports the hypothesis that the C_EA_ clade originated in Burundi (*PP* = 1) and was later exported to the other east African countries where it further spread, establishing new local epidemics (Figures 3 and 4). Estimation of viral movement among countries, obtained by counting the state changes along the tree nodes, points to the role of Burundi as the most important hub of dissemination of this subtype C lineage in east Africa, followed by Tanzania (Table 2). Several migration events of the lineage C_EA_ from Burundi to Ethiopia (*n* = 4), Kenya (*n* = 5), Tanzania (*n* = 8) and Uganda (*n* = 8) were detected, as well as from Tanzania to both Kenya (*n* = 3) and Uganda (*n* = 7). Importation of the C_EA_ lineage into Burundi from other east African countries, and viral exchanges between Ethiopia, Kenya and Uganda were seldom detected in our dataset.

**Table 2:** Estimated number of migration events of HIV-1 C_EA_ clade among east African countries.

From/To	Burundi	Ethiopia	Kenya	Tanzania	Uganda
Burundi	-	4	5	8	8
Ethiopia	0	-	0	0	1
Kenya	0	0	-	1	1
Tanzania	0	0	3	-	7
Uganda	0	0	0	0	-

The Bayesian analysis also supports an important phylogeographic subdivision within the C_EA_ lineage. Consistent with the ML topology (Figure S2), most subtype C sequences from Ethiopia, Kenya, Tanzania and Uganda branched in country-specific monophyletic sub-clusters that most probably (*PP*≥0.93) had a Burundian origin (Fig. 3). The C_ET1_ and C_ET2_ lineages, that correspond to the so called Ethiopian-C clade, comprise 44% of all Ethiopian sequences here included and were almost exclusively composed by sequences from this country. The C_KE_ and C_UG_ lineages comprise 33% and 37% of all sequences from Kenya and Uganda, respectively, and their circulation seems to be mainly restricted to those countries. Finally, the C_TZ_ lineage comprises 39% of all Tanzanian sequences analyzed and has also been disseminated to Kenya and Uganda. Both ML and Bayesian analyses further suggest that the C_EA_ clade branched in two major sub-clades: one composed by sequences from Burundi and lineages C_ET1_, C_ET2_ and C_UG;_ the other one composed by sequences from Burundi and lineages C_KE_ and C_TZ_. The statistical support of such major sub-clades in Bayesian analysis, however, was not significant (*PP*<0.50) and this observation should be interpreted with caution.

### Time-scale of the HIV-1 C_EA_ clade

The median estimated evolutionary rate for the *pol* region of the C_EA_ clade was 1.8×10^−3^ (95% highest posterior density [HPD]: 1.1×10^−3^–2.4×10^−3^) subst/site/year, similar to that previously estimated for HIV-1 subtype C lineages circulating in South America [60] and southern Africa [6]. Importantly, the coefficient of rate variation was higher than zero (0.26 [95% HPD: 0.21–0.31]), thus demonstrating a significant variation of substitution rate among branches in the C_EA_ clade and supporting the use of a relaxed molecular clock model to reconstruct the time-scale of this lineage. According to this analysis the C_EA_ clade started to spread in Burundi at 1962 (95% HPD: 1942–1975), while major sub-clades C_ET1_/C_ET2_, C_KE_, C_TZ_ and C_UG_ began to expand in Ethiopia, Kenya, Tanzania and Uganda, respectively, by the early 1970s (Figure 3).

### Time-scale of the HIV-1 subtype C Ethiopian clades

**Figure 5:**
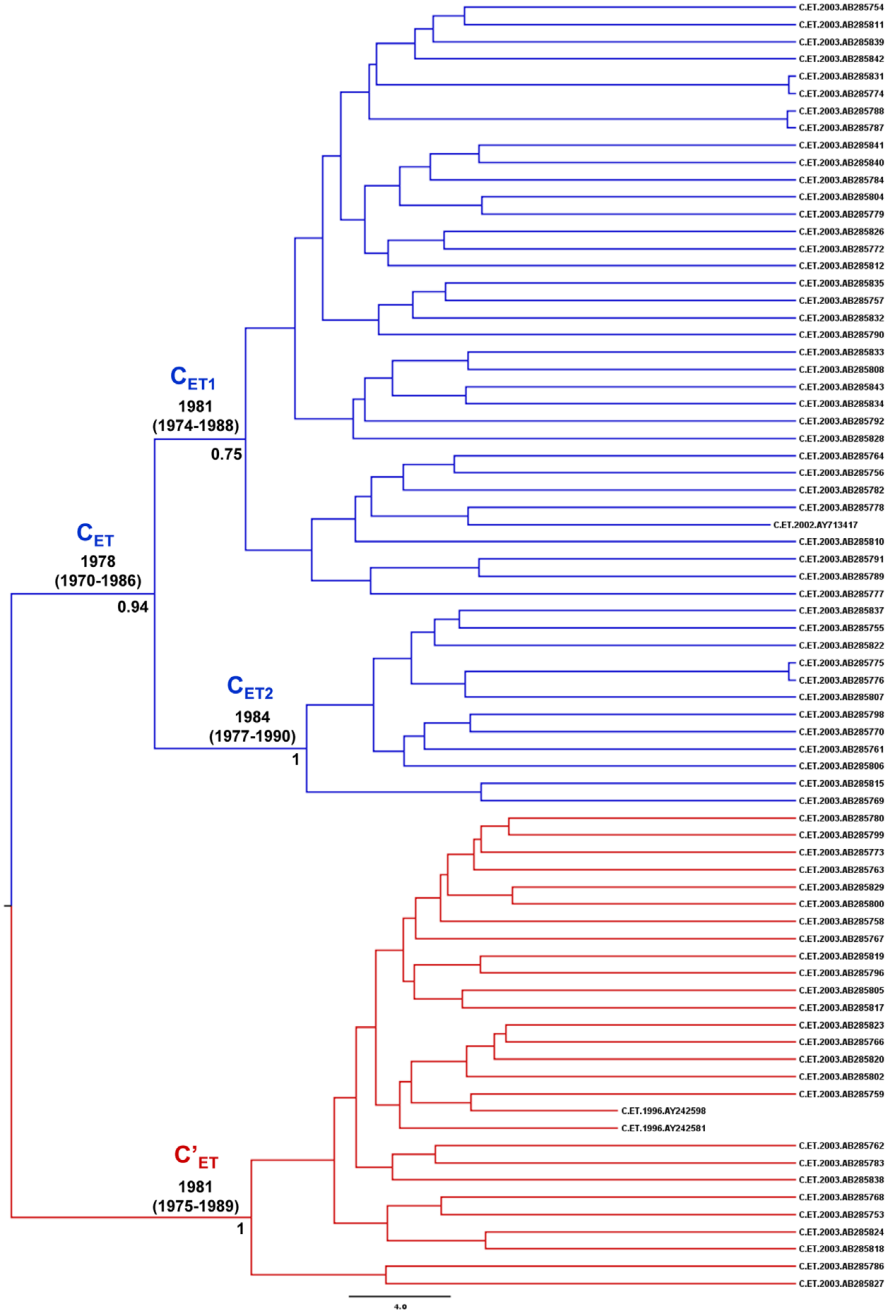
Time-scaled Bayesian MCC tree of major Ethiopian HIV-1 subtype C lineages. MCC tree was obtained after exclusion of putative C/C′ intrasubtype recombinant sequences. Branches are colored according to the initial clade assignment of each sequence based on ML analysis: C_ET_ (blue) and C′_ET_ (red). The *PP* support and the median age (with 95% HPD interval in parentheses) are indicated only at key nodes. Horizontal branch lengths are drawn to scale with the scale at the bottom indicating years. The tree was automatically rooted under the assumption of a relaxed molecular clock.

The time-scale of the two major Ethiopian clades (C_ET_ and C′_ET_) was also estimated by combining all sequences from this country in a single dataset and incorporating the posterior distribution of the substitution rate previously estimated for the C_EA_ lineage as an informative prior. This analysis resulted in a Bayesian MCC tree in which clades C_ET_ and C′_ET_ were poorly supported (*PP*<0.5) and several strains branched outside those major clades (Figure S3). A careful exploration of Ethiopian sequences, revealed that some strains initially classified within clades C_ET_ (*n* = 8) or C′_ET_ (*n* = 10) and those strains that branched outside major Ethiopian clades (*n* = 2) are putative C/C′ intrasubtype recombinant viruses (Figure S3). When those viruses were excluded, the clades C_ET_ and C′_ET_ segregate in two highly supported (*PP*>0.9) reciprocally monophyletic groups (Figure 5). According to this new Bayesian MCC tree, the median *T*_mrca_ was estimated at 1978 for clade C_ET_, 1981 for sub-clade C_ET1_, 1984 for sub-clade C_ET2_, and 1981 for clade C′_ET_ (Figure 5).

## Discussion

This study demonstrates a significant phylogeographic subdivision of HIV-1 subtype C strains circulating in the east respect to those circulating in the central and southern African regions, consistent with a recent study [8]. Most (73%) subtype C sequences from east Africa analyzed in this study branched within a highly supported monophyletic clade, here called C_EA_, that comprise 100% of subtype C sequences from Burundi, 97% from Uganda, 64% from Kenya, 61% from Ethiopia, and 49% from Tanzania. This major east African clade also comprises a minor proportion (<10%) of sequences from central Africa, but no sequence from southern Africa, thus indicating that its circulation is mainly restricted to the east African region. Of note, the genealogies previously inferred for HIV-1 subtypes A and D also support a model of limited introduction of each subtype into east Africa, followed by a subsequent local expansion [61].

Our phylogeographic study suggests that the C_EA_ clade most probably originated in Burundi and after a period of local expansion, this viral lineage was disseminated at multiple times to Ethiopia, Kenya, Tanzania and Uganda, where it generated new local epidemics. Several introductions of the C_EA_ lineage from Tanzania into both Kenya and Uganda were also detected, while viral exchanges between Ethiopia, Kenya and Uganda were less frequent. Five major country-specific monophyletic sub-clusters were detected within the C_EA_ clade that comprise 44%, 33%, 37%, and 39% of all sequences from Ethiopia, Kenya, Uganda and Tanzania here included, respectively. Thus, despite frequent viral movement among east African countries, a significant proportion of subtype C infections in Ethiopia, Kenya, Tanzania and Uganda most likely resulted from the expansion of a few ancestral C_EA_ strains.

It has been suggested that interconnectivity between population centers was a critical factor in the spread of HIV-1 subtypes A and D across Africa [61]. The restricted circulation of the C_EA_ lineage in southern African countries is consistent with this model, considering the relative inaccessibility between the principal population centers of eastern and southern African regions. This model, however, is not consistent with the proposed role of Burundi as the main hub of dissemination of the C_EA_ clade in the region, as this small country is poorly interconnected to other east African countries. Previous studies have also shown a strongly supported phylogenetic relationship between subtype C sequences from Brazil, the UK, Burundi and Kenya; thus indicating that the C_EA_ clade has also been disseminated to South America and Europe [60], [62], [63]. These evidences suggest that factors other than accessibility may have shaped the dissemination of the C_EA_ clade at both local and global scale.

Burundi has known many violent ethnic conflicts mainly since the 1960s that resulted in large migration flows. Two major civil conflicts that took place in Burundi in 1972 and 1993 generated especially large human movements with the former producing around 300,000 refugees and the latter producing about 687,000 [64]. Most refugees initially crossed the border of their country in the east, fleeing to neighboring Tanzania, followed by movement into other neighboring African countries and later to Europe and North America. It has been estimated that there are about 200,000 Burundians currently living in Tanzania, 18,000 in the Democratic Republic of the Congo, 4,000 in Uganda, 10,000 in the European Union, and about 3,000 in the USA and Canada [64]. The molecular clock analysis clade traced the origin of the C_EA_ lineage in Burundi to the early 1960s, while the onset date of the major sub-clades circulating in Ethiopia, Kenya, Tanzania and Uganda was estimated at around the early 1970s, coinciding with the first large Burundian migration flow. These analyses support the notion that the Burundian migration flow occurring in 1972 may have played a fundamental role in the regional and international dissemination of the C_EA_ clade.

While subtype C epidemic in Burundi and Uganda is largely dominated by the C_EA_ clade, a second major subtype C lineage is also circulating in Ethiopia. Our results showed that the two Ethiopian lineages previously designated C and C′ [13], resulted from independent founder strains originated in the eastern and southern African regions, respectively, and further confirmed the circulation of intra-subtype C/C′ recombinants in Ethiopia [40]. The prevalence of C/C′ recombinant viruses estimated in our dataset (20%) was equal to the percentage found in the general Ethiopian population [40]. The onset date of Ethiopian clades C and C′ was dated to between the early 1970s and the early 1980s; consistent with previous estimations [65], [66], [67].

A large collection of minor subtype C lineages of southern African origin were detected in Kenya and Tanzania, which together represent 36% and 51% of sequences from those countries here analyzed, respectively. These lineages seem to have a more restricted expansion than the C_EA_ clade, although they were particularly prevalent (100%) in southwest Tanzania (Mbeya region), close to Zambia and Malawi. The co-circulation of subtype C sequences from both east and southern African origin in Tanzania is consistent with its intermediate geographical position between eastern and southern countries. It is unclear whether subtype C clades of southern African origin detected in Kenya were introduced from Tanzania and/or directly from southern Africa.

It is also unclear the relevance of these findings for HIV-1 vaccine design. Possible correlations of distinct HIV-1 subtype C clades with differential susceptibility to neutralizing antibody and/or cellular immune responses should be explored to justify the selection of vaccines incorporating one or multiple immunogens derived from major African subtype C clades [8]. It is also uncertain whether distinct subtype C lineages may possess different biological properties that affect disease progression and viral transmission. A recent study conducted in Ethiopia showed that infection with clade C_ET_ is associated with initially lower HIV-1 RNA plasma loads but more rapid onset of disease than infections with clade C′_ET_ [68]. The authors proposed that the clade C_ET_ may be less efficiently transmitted than clade C′_ET_, which is consistent with epidemiological evidence that show that the strain C′_ET_ has gained ground and surpassed the clade C_ET_ over time [40], [68]. New studies are necessary to determine if subtype C lineages of east African origin are less transmissible than those originated in southern Africa.

In conclusion, the results presented here point to the existence of a HIV-1 subtype C lineage characteristic of east Africa, which accounts for >70% of subtype C infections in this African region. This lineage probably emerged in Burundi in the 1960s and about 10 years later spread to Ethiopia, Kenya, Uganda and Tanzania, where it disseminated establishing new local epidemics. The subtype C epidemics in Ethiopia, Kenya and Tanzania also resulted from the introduction and dissemination of additional lineages of southern African origin. The explanation for the pattern of spread of the HIV-1 subtype C epidemic in east Africa is probably multifactorial and includes founder effects, massive migration between countries as a consequence of ethnic conflicts and geographical proximity.

## Supporting Information

Figure S1 **Substitution saturation and likelihood mapping analyses.** a) Transition (blue line) and transversion (green line) versus divergence plot for the HIV-1 subtype C *pol* dataset. b) Likelihood mapping of 10,000 random quarters selected from the HIV-1 subtype C *pol* dataset. Distribution (left triangle) and percentage (right triangle) of dots plotted in each region of the map. Each dot represents the likelihoods of the three possible tree topologies for a set of four sequences (quartets) selected randomly from the dataset. The dots localized on the vertices, in the centre and on the laterals represent the tree-like, the star-like and the network-like phylogenetic signals, respectively.
(PPT)

Figure S2 **Close view of the HIV-1 C_EA_ lineage despited in** **Figure 1** **.** The color of branches represents the country from where the sequence originated, according to the legend shown on the left. The boxes highlight the position of the major country-specific sub-clades detected in our study. The aLRT support values are indicated only at key nodes.
(PPTX)

Figure S3 **Time-scaled Bayesian MCC tree of HIV-1 subtype C** ***pol*** **sequences from Ethiopia.** Branches are colored according to the initial clade assignment of each sequence based on ML analysis: C_ET_ (blue), C′_ET_ (red), other clades (green). The *PP* support is indicated only at key nodes. Positions of the putative interclade C/C′ recombinant sequences are marked with asterisks. Horizontal branch lengths are drawn to scale with the scale at the bottom indicating years. The tree was automatically rooted under the assumption of a relaxed molecular clock. Representative bootscanning plots of some putative C/C′ intrasubtype recombinant sequences are depicted on the right. Query sequences were compared to reference sequences of HIV-1 clades A1 (AB253429), D (AY371157), C_ET_ (AY242589), and C′_ET_ (AY242581).
(PPTX)
