# Supplementary material for: Phylodynamics of HIV-1 Subtype C Epidemic in East Africa
Source: PLoS One. 2012 Jul 27;7(7):e41904. doi: 10.1371/journal.pone.0041904 (PMC3407063; doi:10.1371/journal.pone.0041904)
Supplement: Figure S1 — Substitution saturation and likelihood mapping analyses. a) Transition (blue line) and transversion (green line) versus divergence plot for the HIV-1 subtype C pol dataset. b) Likelihood mapping of 10,000 random quarters selected from the HIV-1 subtype C pol dataset. Distribution (left triangle) and percentage (right triangle) of dots plotted in each region of the map. Each dot represents the likelihoods of the three possible tree topologies for a set of four sequences (quartets) selected randomly from the dataset. The dots localized on the vertices, in the centre and on the laterals represent the tree-like, the star-like and the network-like phylogenetic signals, respectively. (PPT) [file pone.0041904.s001.ppt]

## Slide 1
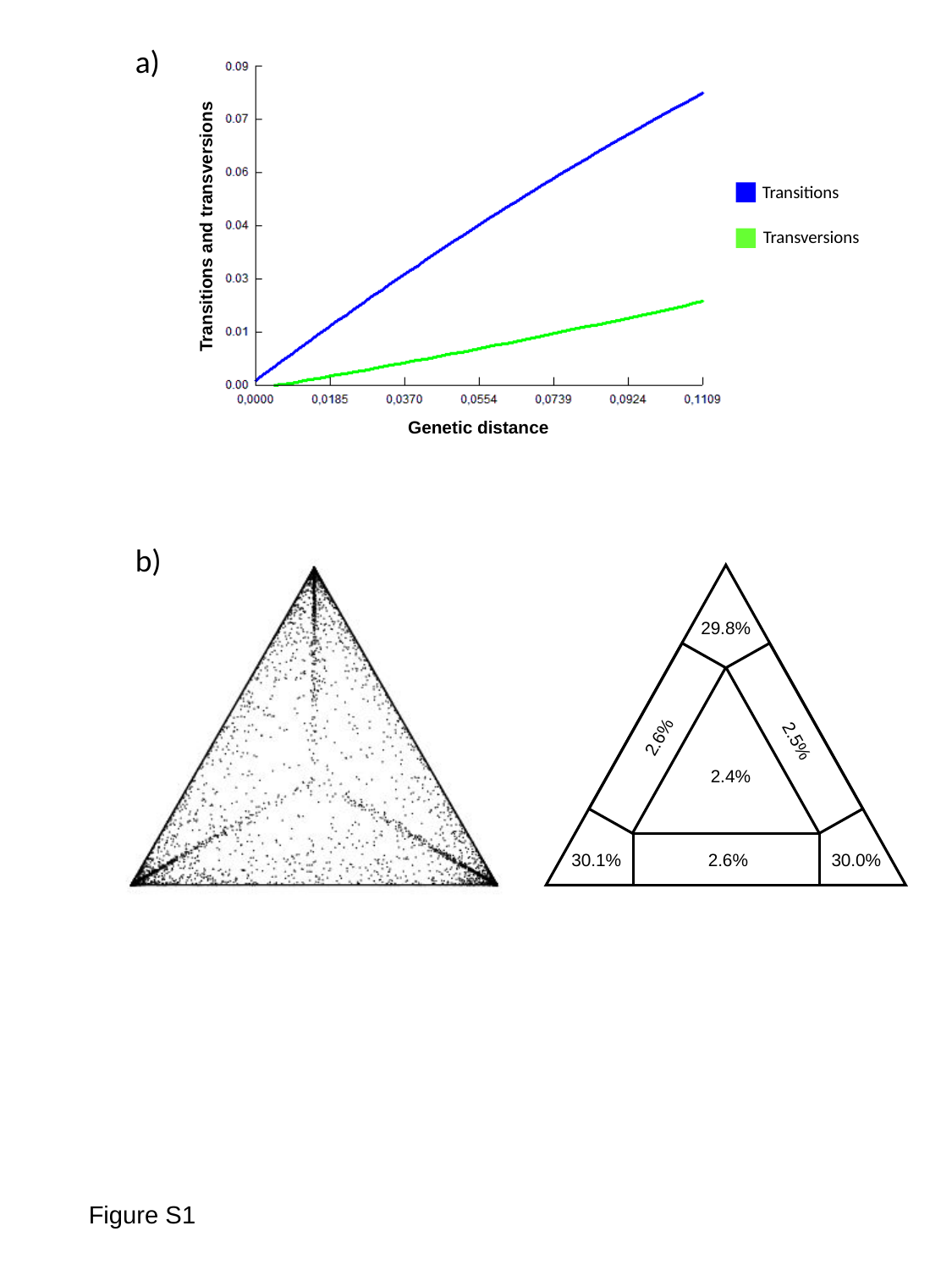

a)
Transitions
Transitions and transversions
Transversions
Genetic distance
b)
29.8%
 2.5%
 2.6%
 2.4%
30.1%
 2.6%
30.0%
Figure S1
